# Detection of foot-and-mouth disease virus RNA using a closed loop-mediated isothermal amplification system

**DOI:** 10.3389/fmicb.2024.1429288

**Published:** 2024-07-31

**Authors:** Natasha Edwards, Julien Reboud, Xiaoxiang Yan, Xin Guo, Jonathan M. Cooper, Jemma Wadsworth, Ryan Waters, Valerie Mioulet, Donald P. King, Andrew E. Shaw

**Affiliations:** ^1^The Pirbright Institute, Woking, United Kingdom; ^2^Division of Biomedical Engineering, School of Engineering, University of Glasgow, Glasgow, United Kingdom

**Keywords:** foot-and-mouth disease, virus, loop-mediated amplification, LAMP, point-of-need, diagnosis, bioengineering

## Abstract

Foot-and-mouth disease (FMD) is a highly contagious viral disease of cloven-hoofed animals responsible for economic losses that amount to >$20 billion annually. Rapid recognition of FMD cases provides vital information to guide control programmes. A range of point-of-need amplification technologies have been developed which allow sensitive detection of the causative virus (FMDV) in the field at locations remote from laboratories. Here we describe a novel system to detect FMDV RNA using loop-mediated isothermal amplification (LAMP). This test was evaluated using a panel of FMDV isolates (*n* = 79) and RNA standards demonstrating capability to amplify viral genome directly from clinical material in the absence of nucleic acid extraction. This extraction-free RT-LAMP assay was transferred to a bespoke closed-system lateral flow test (LFT) that was used in combination with a low-cost hand-held heater. Our results show that the RT-LAMP-LFT assay retains a high level of diagnostic and analytical sensitivity when using direct clinical material, with a limit of detection under 80 copies per reaction. Together, our data support the potential for the use of this assay at the point-of-need to facilitate rapid feedback on the status of suspect cases.

## 1 Introduction

Foot-and-mouth disease (FMD) is a damaging disease of livestock caused by the picornavirus FMD virus (FMDV). FMDV affects both wild and domestic members of the Artiodactyla including cattle, sheep, pigs and buffalo. Infection with FMDV results in acute disease, characterised by fluid-filled vesicles which develop within epithelial tissues, including those of the mouth, feet and teats. Economic losses associated with FMD outbreaks in endemic nations are estimated at US$6.5–21 billion annually, largely as a result of reduced productivity, costs associated with control and the impacts of trade restrictions (Knight-Jones et al., 2017). In contrast, outbreaks of FMD in disease-free nations are estimated to cause losses of over US$1.5 billion (Knight-Jones and Rushton, 2013). Indeed, the economic consequences of the 2001 epizootic of FMD in the United Kingdom are estimated to have been £3.1 billion for the agriculture and food chain (20% of estimated total income from farming in 2001), and losses to the tourism industry of up to £3.2 billion (Thompson et al., 2002).

In both endemic and FMD-free nations, the ability to rapidly detect FMDV can help expedite measures to control the spread of the disease. One way in which it is possible to reduce the time to result is to perform the diagnosis at the point of need (PON), rather than in a centralized laboratory, which requires samples to be shipped (further increasing contamination risks as well as expense). The extensive use of lateral flow tests (LFTs) in the SARS-CoV-2 pandemic has highlighted the possibility and utility of remote testing for viral infections using straightforward assays (Eyre et al., 2023). However, the sensitivity of LFTs is dependent upon the binding affinity of the antibodies used in the formulation of the assay. As a result, many LFTs possess lower analytical sensitivity than real-time PCR, which is often the gold standard for the sensitive and specific detection of pathogens (Ferris et al., 2009, 2010; Dinnes et al., 2022; Eyre et al., 2023). In order to overcome the issues of analytical sensitivity, molecular assays have been developed into simple-to-use PON formats. Mobile and handheld PCR machines have been developed and commercialised; however, PCR chemistries require sophisticated hardware capable of thermal cycling a reaction. Such hardware is typically expensive and is often unsuited to the rigours of use outside of a laboratory. Therefore, other molecular diagnostic chemistries that are potentially more suitable for field use with simpler hardware are being evaluated. These technologies include isothermal amplification methods such as recombinase polymerase amplification (Abd El Wahed et al., 2013), nucleic acid sequence based amplification (Collins et al., 2002; Lau et al., 2006, 2008), helicase dependent amplification (Jingwei et al., 2014), Sherlock (Gootenberg et al., 2017; Kellner et al., 2019), and loop-mediated amplification (LAMP). Of these approaches, LAMP assays offer advantages over real-time PCR but with the potential for similar levels of analytical sensitivity. Another significant benefit of LAMP relative to RT-PCR is the greater tolerance to inhibitory substances, with the result that crude samples, including clinical material, such as homogenised vesicular tissue, can often be used directly as a template for amplification (Waters et al., 2014; Howson et al., 2017b). Combined with the ability to generate simple LFT-based readouts, LAMP represents a molecular test with the attributes to be used at the PON. Multiple reverse transcription LAMP (RT-LAMP) assays have been developed for FMDV with different detection methods, including agarose gels (Li et al., 2009; Shao et al., 2010; Chen et al., 2011a,b; Guan et al., 2013; Ding et al., 2014; Ranjan et al., 2014), fluorescence (Dukes et al., 2006), turbidity (Madhanmohan et al., 2013; Yamazaki et al., 2013), probe-annealing (Bath et al., 2020; Lim et al., 2020), colorimetric (Lim et al., 2018; Zhang et al., 2022) and lateral-flow tests (Waters et al., 2014; Howson et al., 2017a).

In common with RT-PCR, RT-LAMP reactions pose a contamination risk in scenarios where the reaction vessel needs to be opened following the incubation step. As such, closed-tube fluorescence or colorimetric-based assays are preferable as the reaction vessel remains closed compared to agarose gel-based electrophoresis. However, whereas fluorescence-based assays require expensive hardware capable of reading fluorescence levels, colorimetric assays can suffer from subjectivity if the colour change is insufficient (Zhang et al., 2022). In this study we investigated multiple steps of existing FMDV RT-LAMP approaches, including the reaction chemistry, sample type and reagent lyophilisation. Furthermore, we demonstrate the use of LAMP to detect FMDV visually via LFT in a self-contained device which does not require the reaction vessel to be opened.

## 2 Methods

### 2.1 Viruses

Historical RNA samples representing 79 different virus isolates were selected from a bank of viral RNAs held at The Pirbright Institute. In order to generate a panel of samples representative of the FMDV species, isolates were selected to encompass geographical and temporal diversity. Virus samples were selected from the WRLFMD reference collection and stocks were grown in LFBK-αVβ6 cells (LaRocco et al., 2013). Virus supernatants were harvested following the appearance of cytopathic effect, clarified by centrifugation for 5 min at 2100 × *g*, aliquoted and stored at −80°C. All work performed with FMDV was completed in the high containment facilities at The Pirbright Institute.

### 2.2 Clinical samples

The samples used for this study were collected from experimental FMDV infections undertaken in the isolation units at The Pirbright Institute. All experimental procedures were conducted in accordance with The Pirbright Institute’s Animal Welfare and Ethical Review Board (AWERB) and the Home Office Animals (scientific procedures) ACT 1986. Cattle samples were derived from the non-vaccinated control animals used in vaccine efficacy trials. Cattle and pigs were inoculated via intradermal inoculation of the tongue and heel-bulb epithelium, respectively. Cattle were infected with different SAT2 viruses whereas pigs were experimentally infected with O/UKG/34/2001.

4N6FLOQSwabs^®^ (Copan, Brescia, Italy) were used to collect clinical samples from animals showing vesicular disease at 2–3 days post inoculation. Nasal, oral, rectal (pigs only) and lesion swabs were collected from all animals present. The ends of the swabs were snapped off into 2 mL tubes containing 1 mL nuclease-free water. Swab elutes were stored at −80°C until tested.

### 2.3 RNA extraction

Viral RNAs were manually purified from cell culture supernatants or swab elutes using the Qiagen RNeasy Mini Kit (QIAGEN, Hilden, Germany) as per the manufacturer’s instructions and were eluted in 50 μL nuclease-free water.

### 2.4 Real-time RT-PCR

Real-time RT-PCR was used to detect FMDV RNA using a primer and probe set targeting the 3D region of the FMDV genome (Callahan et al., 2002). Each PCR reaction comprised 10 μL of 2x Express One-Step SuperScript qRT-PCR Mix (Invitrogen, Thermo Fisher Scientific, Horsham, United Kingdom), 20 pmols of each primer, 7.5 pmols of FAM labelled TaqMan probe, 0.5 μL of 1:10 diluted ROX reference dye, and 2 μL of RT Express One-Step SuperScript qRT-PCR enzyme mix. A total of 5 μL of the RNA template was then added to the well and mixed gently by pipetting. RT-PCR reactions were run using the ‘fast’ method, consisting of a 15 min holding stage at 50°C, followed by 20 s at 95°C, before 50 cycles alternating between 95°C for 3 s and 60°C for 30 s. Ct values were determined as the point at which the fluorescence reached a threshold of 0.1 ∆Rn.

### 2.5 RT-LAMP

RT-LAMP was performed using previously published primers described by Shao et al. (2010) or Dukes et al. (2006) which both target the 3D RNA polymerase-encoding region of the FMDV genome. A 10× concentration stock mix of oligonucleotides was made containing each of the six primers at the following concentrations: F3 and R3 (2 μM), forwards internal primer (FIP) and backwards internal primer (BIP, 16 μM), and forwards loop (F loop) and backwards loop (B loop) primers (8 μM). Working stocks of 10× oligonucleotide mixes were maintained at 4°C.

RT-LAMP reactions were assembled and run according to the Optigene RT-LAMP protocol. Briefly, mastermixes were prepared where each reaction contained 15 μL of 2× RT-LAMP mix (Optigene Ltd., Horsham, United Kingdom), 2.5 μL of the stock primer mix and 2.5 μL of nuclease-free water. A total 20 μL of the mastermix was distributed into the wells of a 96-well plate with the addition of 5 μL of template RNA. The plate was loaded into an Applied Biosystems 7500 Fast Real Time PCR System (Applied Biosystems, Thermo Fisher Scientific, Horsham, United Kingdom) and run with a customised RT-LAMP run method. The RT-LAMP reactions were incubated for 30 min at 65°C followed by a melting curve analysis, starting at 98°C for 15 s, then 80°C for 1 min, followed by 98°C for 30 s and finishing with 80°C for 15 s. A Time to positive value (Tp) was determined as the point at which the fluorescence generated during the reaction reached the threshold of 1×10^5^ ∆Rn.

Direct RT-LAMP reactions, which do not include an RNA isolation step, were performed in an identical fashion as standard RT-LAMP but used 5 μL swab elute as a sample. Direct RT-LAMP reactions were run using an Agilent Mx3005P Real-Time PCR System (Agilent, Stockport, UK) using the run profile described above.

### 2.6 Lateral flow devices

The RT-LAMP mastermix and incubation steps allowing LFT detection of amplification products was identical to the RT-LAMP reactions run in a thermal cycler except for the substitution of labelled oligos. Two of the standard primers were substituted with primers labelled with either FAM/FITC (FIP) or biotin (BIP). After 30 min incubation at 65°C, the RT-LAMP reaction plate was removed from the heat source and placed on ice to terminate the reaction. 20 μL of the RT-LAMP reaction was added to 80 μL reaction buffer followed by an incubation on ice for 5 min. The tube was opened, and the FITC-Biotin LFT strip (TwistDX, Maidenhead, United Kingdom) was submerged in the reaction for 10 min.

### 2.7 Engineered reaction-LFT devices

The RT-LAMP reaction was onboarded to a custom, closed-system LFT adapted from Reboud et al. (2019). The plastic cartridge was laser cut out of 2 mm thick Poly(methyl methacrylate) (PMMA) sheets. The chambers and slots for inserting the LFT strips were sealed with PCR film. Nuclease free water (60 μL) was added to the buffer loading well, located at the bottom of the device and the chamber sealed with adhesive PCR film. The LAMP reaction components (20 μL RT-LAMP master mix and 5 μL sample) were combined in a microcentrifuge tube and mixed by gentle pipetting. The tube was then briefly centrifuged and the 25 μL reaction was loaded into the LAMP reaction well of the LFT and sealed with film. The LFT was kept in an upright position and incubated for 30 min at 65°C. After the 30 min incubation the LFT was removed from the heat source, inverted so the wells were at the top of the device, and pressure applied to the buffer loading well to pump the reaction through to the paper membrane. The results of the reaction were recorded after 10 min of lateral flow along the membrane.

### 2.8 Smartphone operated heater

The engineered closed system LFTs were incubated using a smartphone-controlled heater (Guo et al., 2021). Although other heat sources such as heat blocks, capable of maintaining a constant temperature of 65°C, are also suitable, they often rely on mains power. The small, hand-held heater is battery powered and connected via Bluetooth to a mobile phone on which a purpose-built app was uploaded. The app was used to set the temperature and duration of the reaction, in addition to monitoring the temperature of the heater as the reaction proceeded. After the command was sent to the heater the LFT was inserted into the heater for the full 30 min duration of the reaction. Once the reaction finished, the LFT was removed from the heater and inverted with pressure applied to the buffer loading well as described above.

## 3 Results

### 3.1 Variation exists between LAMP mastermixes

LAMP reagents are continually being developed and thus assay performance may vary according to the particular reagents that are used. Based upon a previous evaluation (Howson et al., 2017b), we selected the Dukes et al. (2006), henceforth ‘Dukes’ and Shao et al. (2010), henceforth ‘Shao’, FMDV LAMP assays for preliminary appraisal prior to implementation in novel formats.

**Figure 1:**
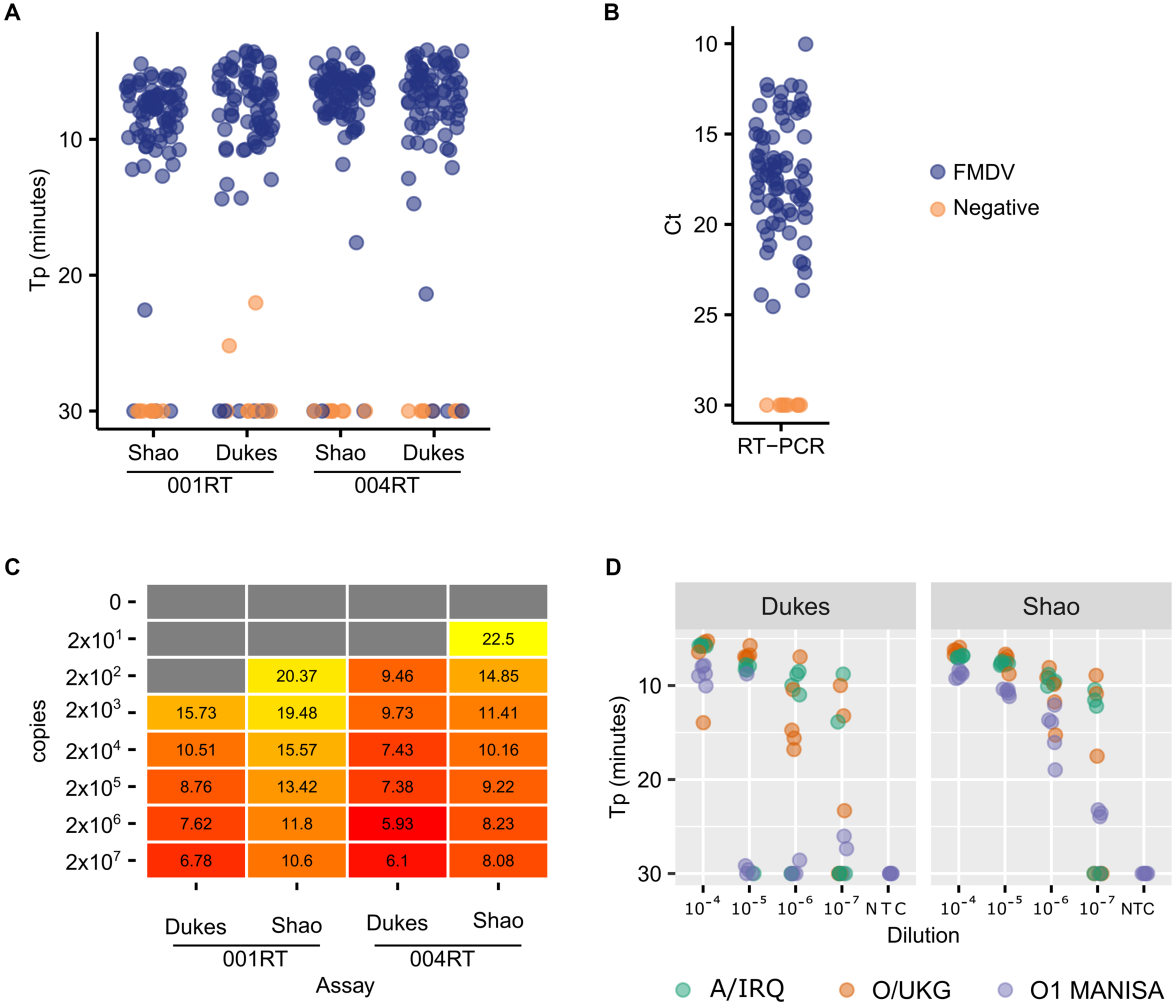
Comparative performance of two FMDV RT-LAMP assays. A total of 79 virus isolates were tested using the Dukes and Shao designs, using two reagent mixes (001RT and 004RT) **(A)**. As a control, the same isolates were also tested using real-time RT-PCR targeting the 3D gene (Callahan et al., 2002) **(B)**. The absolute sensitivity of each assay/reagent combination (copies/reaction) was assessed using *in vitro* transcribed RNA transcripts **(C)**. The analytical sensitivity of the two assays was further evaluated using a decimal dilution series of three FMDV isolates (A/IRQ/24/1964, O/UKG/35/2001, and O1 Manisa), with five replicates tested at each dilution **(D)**.

First, we assessed the performance of the Dukes and Shao oligos with two mastermixes which contained a reverse transcriptase: the 001 RT mix (001RT, used previously in Howson et al., 2017a) and the newer 004 mix (004RT) from Optigene Ltd. (Horsham, United Kingdom). During this reagent selection phase, all of the analyses were performed using real-time detection using an Applied Biosystems 7500 real-time RT-PCR machine. The diagnostic sensitivity of both of these LAMP assays with the two mastermixes was assessed using a panel of nucleic acid samples purified from 79 virus isolates (Figure 1A). The Shao assay (96.2% sensitivity) outperformed the Dukes assay (92.4% sensitivity) when using the 001RT mastermix. Notably, two no template control samples were falsely positive by the Dukes assay using the 001RT mix. In contrast, the diagnostic sensitivity for the assays was equivalent using the 004RT mastermix, with both assays failing to detect three isolates within the 30 min incubation (96.2% sensitivity) and with an approximately equal time to positivity (mean 6.7 and 6.9 min for the Shao and Dukes assays, respectively, Figure 1A). All of the samples tested were positive by real-time RT-PCR (Callahan et al., 2002; Figure 1B).

To accurately determine the analytical sensitivity exhibited by different mastermixes, we used *in vitro* transcribed RNA standards. In all cases, the Dukes assay resulted in lower Tp values (Figure 1C). However, the overall analytical sensitivity was equivalent between the assays, with both assays capable of detecting down to 200 copies of RNA in a decimal dilution series. The 004 reagents outperformed the 001 version, thus all further experiments were performed using the 004RT mix. To further evaluate the assays, dilution series were prepared of O1 Manisa, O/UKG/35/2001 and A/IRQ/24/1964 virus isolate supernatants. In these experiments, the Shao assay outperformed the Dukes assay, where 100% of replicates at the 10^−6^ dilution were amplified before 20 min (Figure 1D). In contrast, only at the 10^−4^ dilution were all of the replicates positive when using the Dukes assay (Figure 1D).

### 3.2 LAMP reactions can be performed in the absence of RNA purification

A distinct advantage of LAMP assays is that they are reported to be more tolerant to the presence of impurities in the sample. As a result, crude samples can often be used in LAMP reactions. Indeed, previous work has shown that it is possible to use diluted epithelial suspension directly added to LAMP reactions for the detection of FMDV (Howson et al., 2017b). However, the preparation and dilution of epithelial suspensions is not an optimal workflow for field use. Here, we evaluated the possibility of using swabs for direct testing by LAMP without prior RNA extraction.

**Figure 2:**
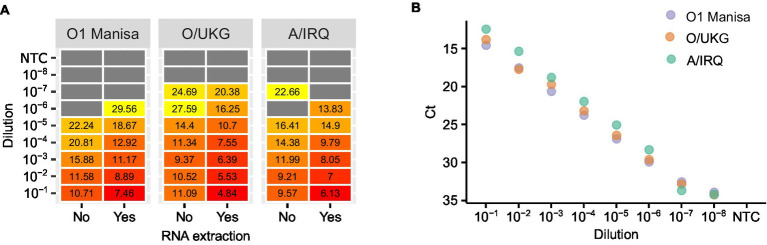
An evaluation of the impact of nucleic acid purification on LAMP reactions. Three FMDV isolates were titrated and either tested directly (‘No’) or tested following RNA extraction (‘Yes’) **(A)**. The extracted RNA was also tested using real-time RT-PCR as a comparison of the relative sensitivities of the two chemistries **(B)**.

First, we compared the impact of adding a dilution series of virus culture supernatants to LAMP mixes either with or without RNA extraction. In parallel, we tested the extracted RNA using real-time RT-PCR. The overall concordance between LAMP reactions using RNA or direct testing of virus supernatants was high, although as expected the inclusion of a purification step increased the analytical sensitivity of the assay (Figure 2A). In the experiments performed using culture supernatant, the LAMP reaction limit of detection was consistently around one log10 less sensitive than real-time RT-PCR (Figure 2B).

**Figure 3:**
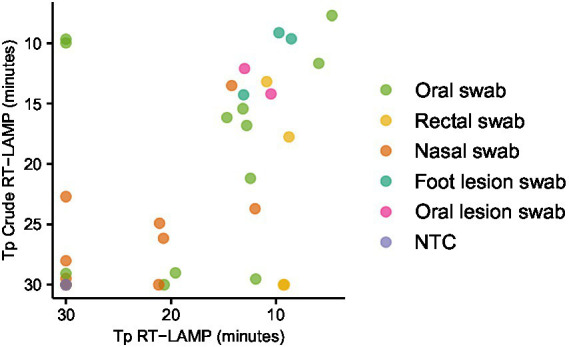
Evaluating the use of clinical swabs as RT-LAMP templates. Swab samples were collected from experimentally infected animals and the swab tip eluted in 1 mL nuclease free water. The swab elute was tested either directly (direct RT-LAMP) or following RNA extraction (RT-LAMP).

In order to establish a practical workflow for on-farm, PON testing, we collected oral, nasal, rectal and lesion swab samples from animals experimentally infected with FMDV, eluted in 1 mL nuclease free water. The water eluate was either tested in LAMP reactions directly (direct RT-LAMP) or tested following RNA extraction (Figure 3). The use of the crude elute as a template in the LAMP reactions was found to compromise the assay sensitivity. Only a weak positive relationship was observed between the Tp for extracted RNA and the Tp for direct RT-LAMP, indicating a stochastic level of inhibition of the LAMP assay. However, lesion swabs consistently gave positive results both with and without prior RNA extraction (Figure 3).

### 3.3 Closed platform

**Figure 4:**
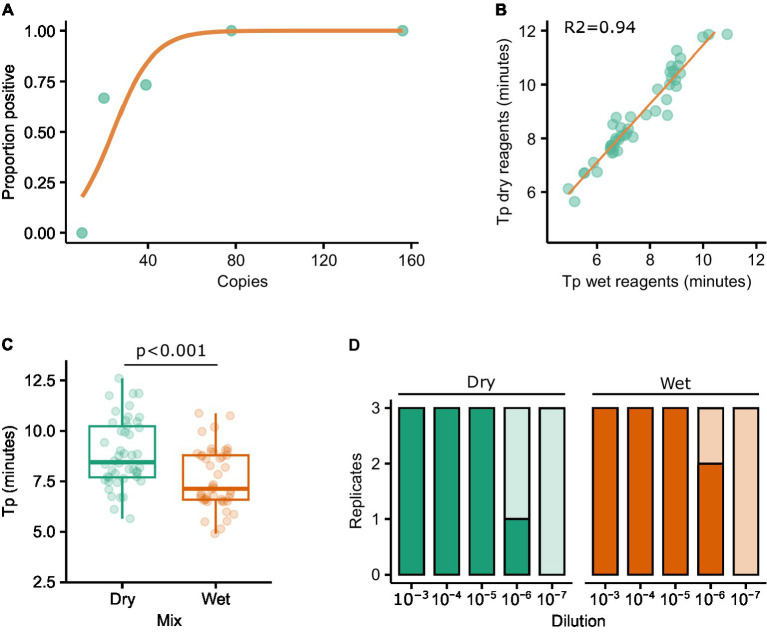
Probit analysis was performed to determine the sensitivity of the RT-LAMP reactions when using a lateral flow output **(A)**. The impact on sensitivity as a result of using lyophilised/resuspended reagents was assessed by testing a panel of FMDV isolates with both the ‘wet’ and resuspended lyophilised (‘dry’) reagents. Tp results correlated well between the reagents (*R*^2^ = 0.94) **(B)**, although the median Tp was significantly different (*T*-test *p* < 0.001) **(C)**. Similar levels of sensitivity were observed using diluted FMDV O1 Manisa RNA **(D)**.

The LAMP mastermixes utilised in this study include a fluorescent dye which binds to double stranded DNA. The inclusion of a dye allows amplification to be monitored in real-time using a real-time RT-PCR machine. However, a strength of LAMP is that it is isothermal and thus requires simpler hardware, in turn making it more amenable to PON testing. The addition of FITC or biotin to the oligos used in the assay allows the result of the reaction to be assessed using lateral flow strips, thus abrogating the need for expensive hardware capable of detecting fluorescence. First, we performed a probit analysis to evaluate the performance of LAMP reactions using labelled oligos in combination with lateral flow strips. Replicate LAMP reactions (*n* = 15) were assembled independently at specific dilutions of RNA and, following incubation, all of the reactions were assessed for amplification (‘yes’/‘no’) by running each reaction on a lateral flow strip. The proportion of reactions at each dilution that were positive was then calculated and plotted (Figure 4A). Using labelled oligos with a lateral flow strip output it was possible to consistently detect 80 copies of RNA. Furthermore, 70% of reactions were positive when 20 copies of RNA were added to the reaction (Figure 4A).

Next, we explored the impact of lyophilising the reagents in such a way that a cold chain would not be necessary. To test this, the wet and dry reagents were trialled using the original panel of 79 RNAs used to compare the assays, with a good relationship observed between the two sets of reagents (*R*^2^ = 0.94, Figure 4B). However, the Tp was significantly longer when using dried (media*n* = 8.46) vs. wet (media*n* = 7.14) reagents (Figure 4C, *p* < 0.001). A dilution series of O1 Manisa RNA was prepared in triplicate and tested directly in ‘wet’ reagents as per previous experiments, or in rehydrated LAMP mixes (‘dry’) and run on LFTs. Broad concordance between wet and dry reagents was observed, with 2/3 and 1/3 reactions positive at the 10^−6^ dilution using wet and dry reagents, respectively (Figure 4D). To further evaluate the performance of dried vs. wet reagents in a LFT format, we tested three PCR-positive lesion swabs derived from experimentally infected pigs by adding elutes from the swabs directly to the LAMP reactions. In all cases, both the wet and dry reagents produced positive LFT results (data not shown).

**Figure 5:**
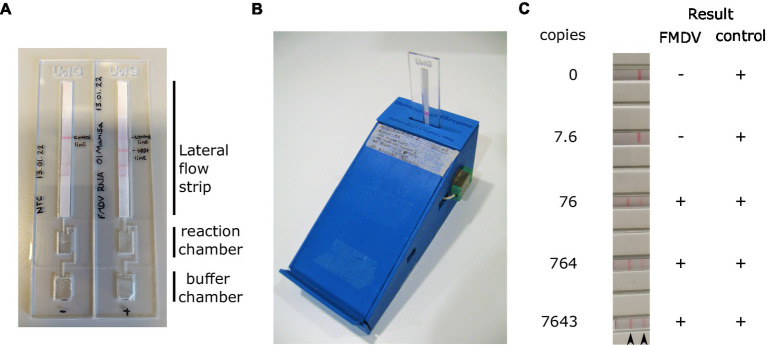
RT-LAMP reactions were assembled in the reaction chamber the devices **(A)**. Prior to sealing, running buffer was also added to the buffer chamber. Sealed RT-LAMP reactions were run using a bespoke Bluetooth controlled heater **(B)**, before pressing the buffer chamber to push the reaction onto the loading pad of the lateral flow strip **(A)**. A dilution series of *in vitro* transcribed RNA was used to assess the analytical sensitivity of the LAMP test using this device. Positive results were always obtained for the control band **(C)**. Arrowheads indicate the position of the FMDV test line (left) and control band (right).

The necessity to open LAMP reaction vessels following amplification for visualisation of the result on the LFT poses a significant risk of contamination. To overcome this, we assessed the performance of closed devices incorporating an amplification chamber, a buffer chamber and a FITC-biotin LFT strip (Figure 5A). These prototype disposable devices produced by the University of Glasgow (UK) can be incubated using a portable heater controlled via a mobile phone app (Figure 5B). The devices were loaded with buffer and LAMP reaction mix, and a dilution of RNA template added to the reaction chamber. The device was sealed using PCR film and incubated at 65°C for 30 min. Upon completion of the incubation, the buffer chamber was squeezed such that the reaction and LFT buffer were mixed and pushed onto the lateral flow strip. The LFT was incubated for 10 min after which the result was read visually. In line with previous experiments, the limit of detection was below 80 copies per reaction (Figure 5C).

## 4 Discussion

FMDV can spread rapidly, and the time taken to obtain a diagnosis is a critical parameter in the deployment of effective control measures. Rapid confirmation of FMDV presence is particularly relevant in countries for which the virus is exotic, as control strategies rely upon the prompt recognition of cases so that control measures can be quickly implemented to minimise onward transmission. Laboratory based molecular assays have greatly reduced the time required to obtain a positive result, in comparison to traditional virus isolation methods. However, laboratory diagnosis requires samples to be transported to laboratories with suitable levels of containment, potentially resulting in delays or samples being compromised. As a consequence, molecular tests that permit FMDV detection by PCR away from the laboratory have been developed (Howson et al., 2018; Edge et al., 2022; Stanhope et al., 2022). However, PCR detection is complex and expensive as thermocycling and RNA extraction are required. In contrast, isothermal assays such as LAMP only need a single incubation temperature and previous studies have demonstrated that LAMP is a suitable technology to fill this field-based detection niche (Howson et al., 2017b).

Multiple LAMP assays have been designed and optimised for the detection of FMDV. However, these approaches often still include aspects which would be restrictive for field deployment, including the requirement for a cold chain, expensive equipment, or the need to open tubes post amplification. Here, we evaluated multiple aspects of the LAMP process to facilitate the deployment of this technology in decentralised locations. The specificity of nucleic acid based molecular assays such as LAMP is defined by the target oligonucleotide sequences. The Shao and Dukes assays both target the 3Dpol coding sequence within the P3 region of the genome. In contrast, the serotype of FMDV is determined by the outer capsid proteins VP1, VP2, and VP3, encoded within the P1 region of the genome. As such, serotype has minimal relevance when testing assays based within the P3 region, therefore it was deeded unnecessary to evaluate every serotype. Similarly, previous studies have confirmed the FMDV-specific nature of the Shao and Dukes oligos (Dukes et al., 2006; Shao et al., 2010).

Having trialled and selected the optimum LAMP reagents, we developed a protocol that could be used to detect FMDV without a nucleic acid extraction step. This work builds on previous studies that have demonstrated that LAMP reactions can be performed in the absence of, or with only basic sample preparation (Howson et al., 2017b, 2018; Reboud et al., 2019). In common with previous studies, we demonstrated that simple swab samples eluted in nuclease-free water represent a suitable sample type for FMDV detection. However, a decrease in analytical sensitivity was observed in the absence of RNA purification, indicating that the incorporation of a nucleic acid isolation step enhances sensitivity, most likely due to the removal of enzymatic inhibitors and/or concentration of the sample. Interestingly, there was little correlation between the Tp values obtained using crude and purified templates, suggesting that the inhibition is largely stochastic. Nevertheless, despite the decrease in analytical sensitivity, the titre of virus within a typical fresh lesion swab (<2 days) is within the limit of detection of the direct RT-LAMP assay.

Previous attempts have been made to address the difficulty of reliably detecting amplification of FMDV by RT-LAMP without the need for complicated/expensive equipment (Yamazaki et al., 2013; Waters et al., 2014). Monitoring the precipitation of magnesium pyrophosphate during LAMP reactions allows the amplification to be assessed based upon the increase in turbidity, allowing more basic equipment to be used (Yamazaki et al., 2013; Shirato, 2019). Similarly, RT-LAMP assays which change colour upon amplification have been developed for the detection of viruses, including FMDV (Lim et al., 2018; Zhang et al., 2022). An alternative approach, as used here, uses labelled oligonucleotides allowing LAMP products to be visualised using a simple LFT, requiring no hardware to visualise the result. Whilst observing a LFT band is ultimately subjective, the contrast against a white background makes visualisation more straightforward. Traditionally the use of LFTs to determine the outcome of molecular methods is severely compromised by the need to open the reaction tube post amplification, introducing a significant risk of contamination. Therefore, a major development in the deployment of simple LAMP assays in the field is the engineering of hardware that is able to generate a visible readout without opening the reaction vessel following amplification. In this study we successfully engineered devices which were capable of both acting as a reaction vessel whilst also incorporating the simplicity of a LFT read-out (Reboud et al., 2019; Witkowska McConnell et al., 2021). We observed that the sensitivity of the closed-tube devices was equivalent to using individual PCR tubes followed by opening the tubes and testing the reaction using commercial FITC-biotin LFTs, with a detection limit of under 100 copies per reaction. Two further adaptations of the setup described here would be beneficial. First, it will be useful to include an in-built positive control (as described by Bath et al. (2020)) which could be incorporated as a third band on an LFT. The use of a positive control is particularly important in the case of direct LAMP assays where the nucleic acid purification step is eliminated and inhibitors or RNases are likely to be present at unknown concentrations. Indeed, the data obtained in the current study showed no relationship between the Tp values of the same sample tested with and without RNA purification, implying that reactions were inhibited in a random fashion. A second way in which to further simplify this system is to dry down the custom reagents in the reaction chambers of the devices, such that it simply requires rehydration with a buffer/sample (Howson et al., 2017a).

In summary, in the study described here we have advanced multiple aspects of the LAMP testing process to make it more readily deployable under field conditions. We have demonstrated that it is possible to use simple swab samples without the requirement for RNA isolation, although further testing of lesion swabs will provide greater confidence with regards to the suitability of this approach. Most importantly, we have developed and tested the utility of custom cartridges capable of both incubating as well as analysing reactions with a LFT readout. Together with the ability to use lyophilised reagents, these approaches enhance the deployability of LAMP testing in the field using simple equipment.

## Data availability statement

The raw data supporting the conclusions of this article will be made available by the authors, without undue reservation.

## Ethics statement

The animal study was approved by the Animal Welfare and Ethical Review Board (AWERB), The Pirbright Institute. The study was conducted in accordance with the local legislation and institutional requirements.

## Author contributions

NE: Investigation, Writing – original draft, Writing – review & editing. JR: Conceptualization, Funding acquisition, Writing – review & editing. XY: Resources, Writing – review & editing. XG: Resources, Writing – review & editing. JC: Writing – review & editing. JW: Resources, Writing – review & editing. RW: Resources, Writing – review & editing. VM: Writing – review & editing. DK: Funding acquisition, Writing – review & editing. AS: Conceptualization, Project administration, Supervision, Visualization, Writing – original draft, Writing – review & editing.
